# webTWAS 2.0: update platform for identifying complex disease susceptibility genes through transcriptome-wide association study

**DOI:** 10.1093/nar/gkae1022

**Published:** 2024-11-11

**Authors:** Chen Cao, Mengting Shao, Jianhua Wang, Zhenghui Li, Haoran Chen, Tianyi You, Mulin Jun Li, Yijie Ding, Quan Zou

**Affiliations:** Key Laboratory for Bio-Electromagnetic Environment and Advanced Medical Theranostics, School of Biomedical Engineering and Informatics, Nanjing Medical University,101 Longmian Ave, Nanjing, Jiangsu 211166, China; Key Laboratory for Bio-Electromagnetic Environment and Advanced Medical Theranostics, School of Biomedical Engineering and Informatics, Nanjing Medical University,101 Longmian Ave, Nanjing, Jiangsu 211166, China; Department of Systems Pharmacology and Translational Therapeutics, University of Pennsylvania—Perelman School of Medicine, 421 Curie Blvd, Philadelphia, PA 19104, USA; Key Laboratory for Bio-Electromagnetic Environment and Advanced Medical Theranostics, School of Biomedical Engineering and Informatics, Nanjing Medical University,101 Longmian Ave, Nanjing, Jiangsu 211166, China; Key Laboratory for Bio-Electromagnetic Environment and Advanced Medical Theranostics, School of Biomedical Engineering and Informatics, Nanjing Medical University,101 Longmian Ave, Nanjing, Jiangsu 211166, China; Department of Pharmacology, School of Basic Medical Sciences, Tianjin Medical University, 22 Qixiangtai Road, Tianjin 300203, China; Department of Pharmacology, School of Basic Medical Sciences, Tianjin Medical University, 22 Qixiangtai Road, Tianjin 300203, China; Yangtze Delta Region Institute (Quzhou), University of Electronic Science and Technology of China, 1 Chengdian Road, Quzhou, Zhejiang 324003, China; Yangtze Delta Region Institute (Quzhou), University of Electronic Science and Technology of China, 1 Chengdian Road, Quzhou, Zhejiang 324003, China

## Abstract

Transcriptome-wide association study (TWAS) has successfully identified numerous complex disease susceptibility genes in the post-genome-wide association study (GWAS) era. Over the past 3 years, the focus of TWAS algorithms has shifted from merely identifying associations to understanding how single nucleotide polymorphisms (SNPs) regulate gene expression, with a growing emphasis on incorporating fine-mapping techniques. Additionally, the rapid increase in GWAS summary statistics, driven largely by the UK Biobank and other consortia, has made it essential to update our webTWAS resource. To address these challenges and meet the growing needs of researchers, we developed webTWAS 2.0, an updated platform for identifying susceptibility genes for human complex diseases using TWAS. Additionally, webTWAS 2.0 provides an online TWAS analysis tool that simplifies conducting TWAS analyses. The updated resource includes 7247 GWAS summary statistics covering 1588 complex human diseases from 192 publications. It also incorporates multiple TWAS methods, such as sTF-TWAS, 3′aTWAS and GIFT, along with an updated interactive visualization tool that allows users to easily explore significant associations across different methods. Other upgrades include a personalized online analysis tool for user-submitted GWAS data and a refined search function that makes it easier to identify relevant associations and meet diverse user needs more efficiently. webTWAS 2.0 is freely accessible at http://www.webtwas.net.

## Graphical Abstract

**Graphical Abstract:**
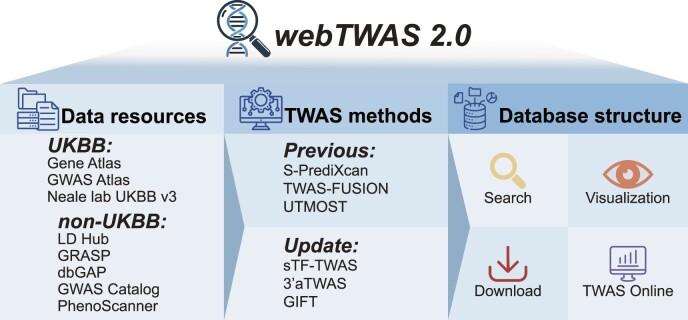

## Introduction

Transcriptome-wide association study (TWAS) was first introduced in 2015 (1), and is a powerful method for integrating genetic variation with gene expression to identify susceptibility genes for complex human diseases. TWAS uses a reference panel to train the weights between whole-genome sequences and gene expression, followed by independent datasets to predict the genetically regulated expression (GReX) component. The GReX is then associated with traits to identify susceptibility genes related to human complex diseases. TWAS addresses several limitations of genome-wide association study (GWAS), including difficulties in interpreting significant signals, as many are located in noncoding regions or result from linkage disequilibrium (LD). Additionally, TWAS significantly improves the power of association studies by more effectively aggregating genetic variants.

The traditional TWAS framework involves two key steps. The first is the training phase, where the relationship between genotype and transcriptome is modeled to determine GReX. The second step associates GReX with phenotypic traits in the target genotype data. In the initial development of TWAS, most efforts focused on improving the accuracy of predicted transcriptomes including PrediXcan (1), TWAS-FUSION (2), UTMOST (3), TIGAR (4) and kTWAS (5), among others (6–10). Over the past 3 years, the focus of TWAS algorithms has shifted toward investigating how SNPs regulate gene expression and identifying causal genes through methods such as fine mapping. This includes integrating prior biological knowledge, such as transcription factors binding to *cis-*regulatory elements (11), pathway networks (12), transcript isoforms (13), 3′ untranslated region alternative polyadenylation (14) and single-cell transcriptome data (15), among others (16–20). These improvements have significantly improved the power of TWAS to identify disease-associated genes. As a result, webTWAS needs to be updated to incorporate these cutting-edge methods and offer researchers a more powerful and accurate platform for gene–trait association analysis.

The aforementioned TWAS methods have been successfully applied to various complex human diseases, including mental disorders (1,3,21), cardiovascular diseases (22–24) and cancers (25–27), among others (20,28–30). These studies highlight the significant potential of TWAS in identifying disease-related susceptibility genes and unraveling the genetic mechanisms underlying complex diseases. Despite the growing interest in TWAS, only three related databases have been developed: TWAS-hub (http://twas-hub.org/), webTWAS (31) and TWAS Atlas (32) have been established to collect and restore TWAS-related datasets and gene–trait associations. TWAS-hub, the first TWAS database introduced in 2018, contains 75 951 gene–trait associations across 342 disease and nondisease traits. webTWAS, based on 1298 curated GWAS datasets, implements three TWAS tools—S-PrediXcan, TWAS-FUSION and UTMOST—and includes 235 064 gene–trait associations across 887 human diseases. TWAS Atlas manually reviewed 200 curated TWAS application publications on human traits, collecting 401 266 gene–trait associations along with online search and visualization tools. These databases offer valuable resources for studying complex human traits using TWAS. However, there are limitations in these TWAS-related databases. TWAS-hub provides analysis results from only one TWAS method, TWAS-FUSION, and has not been updated since 2019. TWAS Atlas collects gene–trait associations from 200 published papers but does not offer online TWAS analysis or results based on GWAS summary statistics using multiple state-of-the-art TWAS methods. webTWAS includes 1298 GWAS datasets and three TWAS methods. With the rapid increase in available GWAS summary statistics, the gradual release of large cohorts such as the UK Biobank (33) (UKBB), and the growing number of TWAS methods, there is an urgent need to update webTWAS. This update includes more GWAS summary statistics and TWAS methods to provide a comprehensive TWAS resource database and an online computational platform. Therefore, it is essential to update webTWAS with more GWAS summary statistics data and to recalculate the disease-susceptible genes using updated TWAS algorithms.

To address these needs, we have updated webTWAS from version 1.0 to 2.0, adding 7247 GWAS summary statistics across 1588 human complex diseases from 192 publications and implementing six representative TWAS methods for analysis. webTWAS 2.0 now restores 661 396 gene–trait associations, covering 1588 diseases, along with detailed disease-relevant tissue, gene and disease information. We addressed that diseases are tissue-specific and identified the most relevant tissue for each disease. Additionally, we developed an interactive visualization tool that allows users to easily explore gene–trait associations, along with an online TWAS analysis tool that provides free access to conduct TWAS analyses. All data in webTWAS 2.0 can be freely searched and downloaded, offering researchers a valuable resource for gene-disease associations. The complete information and resources in webTWAS 2.0 are available at http://www.webtwas.net/.

### webTWAS version 2.0 design

**Figure 1.**
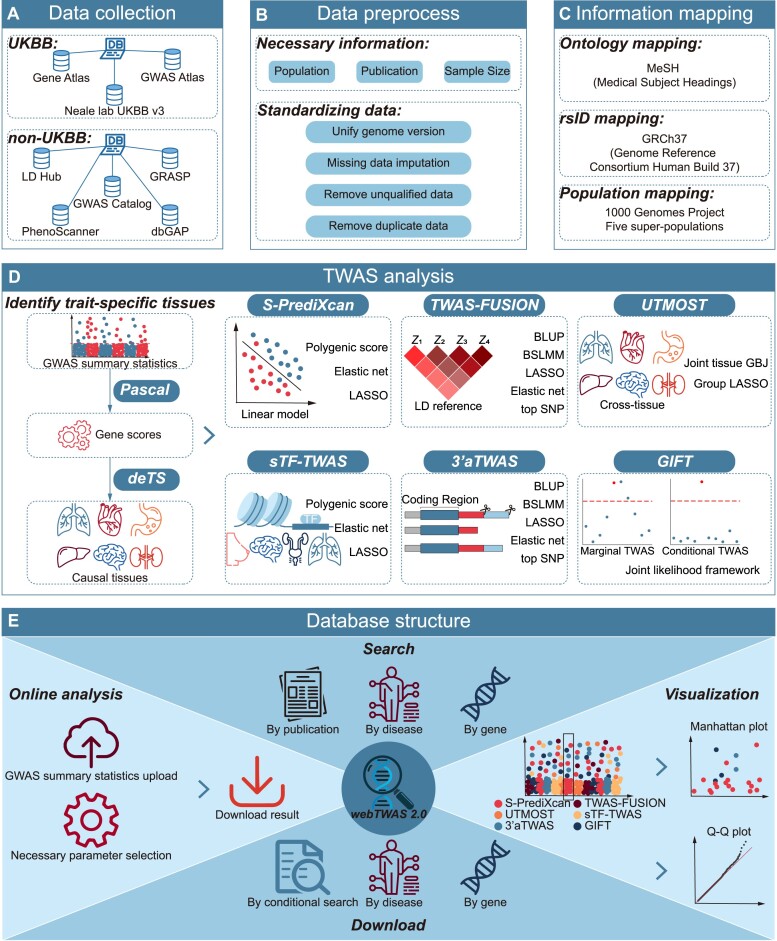
Overview of webTWAS 2.0. (**A**) GWAS summary statistics were collected from two main sources and subjected to preprocessing (**B**) to ensure data quality. (**C**) The datasets were then standardized through information mapping. (**D**) Six TWAS methods were employed for the analysis of the GWAS summary statistics. (**E**) The overall structure of the webTWAS 2.0 database.

Here, we present webTWAS version 2.0, a comprehensive database for disease susceptibility genes identified by multiple TWAS methods. Our platform offers gene–trait associations across a range of complex human diseases and provides an easy-to-use online TWAS analysis tool (Figure 1). Publicly available GWAS summary statistics were collected (Figure 1A), subjected to quality control (Figure 1B), and standardized to ensure clarity and avoid misinterpretation (Figure 1C). For TWAS analysis, six representative methods, including S-PrediXcan (27), TWAS-FUSION (2), UTMOST (3), sTF-TWAS (11), 3′aTWAS (14) and GIFT (17), were employed (Figure 1D). An important feature of webTWAS 2.0 is its ability to account for tissue specificity, enabling researchers to focus on the most relevant tissues for each disease for gene–trait association analysis. Additionally, a user-friendly interface was developed to support the main functions of webTWAS 2.0, including search, online analysis, visualization and data download (Figure 1E). Detailed gene–trait associations for any disease and gene can be easily accessed. The search interface was specifically designed to facilitate quick access to associations of interest. All data in webTWAS 2.0 can be freely downloaded from the database website.

## Updates and new features

### GWAS summary statistics update and pre-processing

The updated webTWAS 2.0 contains a total number of 7247 GWAS summary statistics for various diseases which were used to conduct multiple TWAS analyses. Following the collection criteria outlined in our previous work, CAUSALdb (34) and webTWAS (31), the public GWAS summary statistics were primarily sourced from two categories: UKBB and non-UKBB (Figure 1A). The UKBB GWAS summary statistics were obtained from Neale Lab UKBB v3 (http://www.nealelab.is/uk-biobank), Gene ATLAS (35) and GWAS ATLAS (36). These three sources differ in sample selection, quality control processes and types of association models. For non-UKBB GWAS summary statistics, data were collected from multiple public databases, including GWAS Catalog (37), LD Hub (38), GRASP (39), PhenoScanner (40), dbGaP (41), PGC (https://pgc.unc.edu), MAGIC (http://www.magicinvestigators.org), SSGAC (https://www.thessgac.org) and JENGER (http://jenger.riken.jp/en).

**Figure 2.**
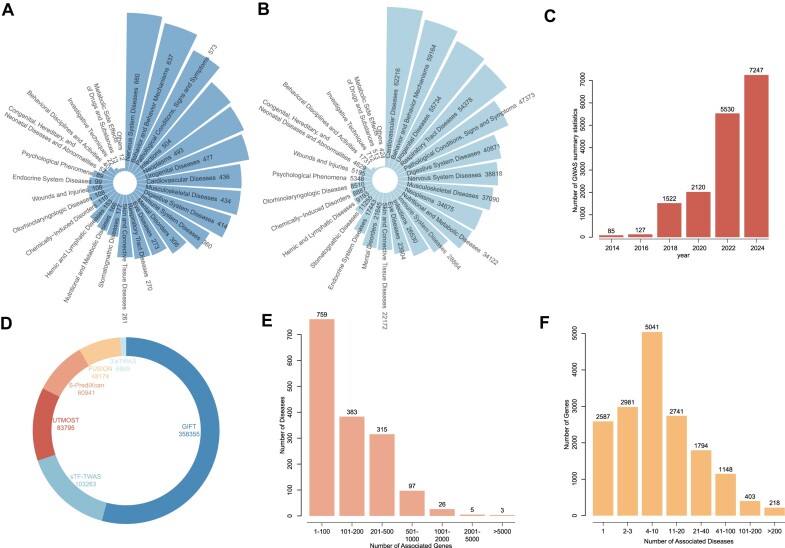
Statistical overview of webTWAS 2.0. (**A**) Number of mapped MeSH terms categorized by disease type. (**B**) Number of gene–disease associations for each disease type. (**C**) Total number of GWAS summary statistics collected by year. (**D**) Distribution of gene–disease associations identified by each TWAS method. (**E**) Distribution of associated genes per disease. (**F**) Distribution of associated diseases per gene.

Quality control was conducted on these GWAS summary statistics. First, for each dataset, relevant information, including sample size and population-related details, was extracted from the original publication. Datasets lacking this information were excluded. For duplicate datasets collected from different sources, only the dataset with the most comprehensive information was retained. Specifically, GWAS summary statistics with unqualified columns, such as those missing standard rsIDs or beta values necessary for computation, were excluded. Missing data, such as *Z*-values, were imputed, and rsIDs and genomic coordinates were standardized to the GRCh37 version. For the trait information of each GWAS summary statistic, we manually mapped each reported trait extracted from the original publication to the medical subject headings (MeSH) term to ensure standardization and prevent misunderstanding. Regarding the population information, we used the five superpopulations defined by the 1000 Genomes Project (42) and, limited by the reference panel of TWAS analysis, retained only GWAS summary statistics of European ancestry (EUR) for analysis. Overall, webTWAS 2.0 contains a total number of 7247 GWAS summary statistics related to 27 complex human disease types and 192 publications (Figure 2A–C).

### Newly integrated TWAS methods for analyzing GWAS summary statistics

With the rapid development of TWAS methods over the past decade, >20 methods have been introduced to identify trait-associated genes using various biological assumptions and different mathematical models. In webTWAS 2.0, we applied six popular and representative methods, including S-PrediXcan (27), TWAS-FUSION (2), UTMOST (3), sTF-TWAS (11), 3′aTWAS (14) and GIFT (17). To ensure the accuracy of TWAS analysis and prevent the misuse of unmatched tissue reference panels—which could affect results due to the tissue-specific feature of gene expression—we employed PASCAL (43) and deTS (44) algorithms to identify disease-specific tissues for each GWAS summary statistics (Figure 1D). First, PASCAL calculated disease-related gene scores, selecting those with *P*-value <0.05. Subsequently, deTS performed a chi-square association test on the disease-related gene set for each GWAS dataset to identify significantly related tissues. For TWAS analysis, we selected the top three tissues for each GWAS summary statistic to determine the appropriate reference panel for the TWAS method parameter settings.

S-PrediXcan computes PrediXcan results using GWAS summary statistics, utilizing elastic net-based models on GTEx v8 release data (45) for each GWAS summary statistics analysis. For TWAS-FUSION, gene weights from GTEx v8 multitissue expression data (http://gusevlab.org/projects/fusion/) are used, with the best model selected from BLUP, BSLMM, LASSO and Elastic Net, and top SNP within the default setting. For UTMOST, association tests are conducted on the top three tissues for each GWAS summary statistic, and gene–trait associations across these tissues are combined using the joint GBJ test with imputation models jointly trained on GTEx data provided by UTMOST. Additionally, two TWAS methods representing different biological assumptions, including transcription factors and 3′ untranslated region alternative polyadenylation were used to calculate gene–trait associations. For sTF-TWAS, weights were provided for four tissues (breast, lung, prostate and brain), and gene–trait associations were calculated for each GWAS summary statistics based on these tissues. For 3′aTWAS, 3′aQTLs from 49 tissues in the GTEx v8 cohort were used (46), with the top three tissues of each GWAS summary statistics selected for the association test. For GIFT, genome-wide eQTL summary statistics from GEUVADIS data provided by GIFT (17) and the two-stage version of GIFT were used to conduct the association tests. For each TWAS method, the default LD matrix provided by the respective method, all sourced from the 1000 Genomes Project (42), was used. The adjusted significance threshold was set at 0.05 divided by the total number of computed genes for each method, and default parameters were applied for all TWAS methods.

After performing TWAS analysis with these methods, a total of 661 396 significant associations were identified. The number of significant associations detected by each method is illustrated in a pie chart in Figure 2D. Specifically, the GIFT method identified the most associations (358 355), and the average number of associations per method was 110 232. For each disease, the average number of associated genes was 208.90. The detailed distribution of associated genes per disease is illustrated in Figure 2E. Conversely, for each gene, the average number of associated diseases was 19.65, with the detailed distribution of associated diseases per gene shown in Figure 2F.

### Updated interactive visualization tool for assessing gene–trait associations

**Figure 3.**
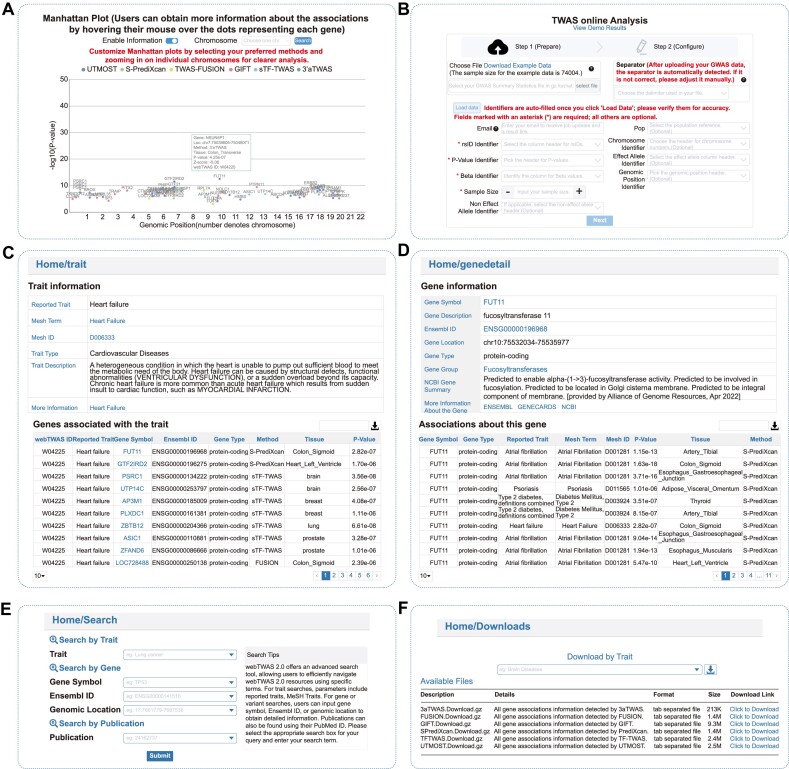
Main pages in webTWAS 2.0. (**A**) Interactive visualization tool displaying detailed gene-disease associations for a selected disease. (**B**) Online analysis tool allowing users to perform customized TWAS analyses. (**C**) Trait page example for heart failure (webTWAS ID W04225). (**D**) Gene page example for the gene FUT11. (**E**) Search page offers three search options: traits, genes or publications. (**F**) Download page provides two types of downloadable files.

To facilitate user access to disease or gene associations of interest, an updated interactive visualization tool was added. On the detailed disease information page, a Manhattan plot displays all associations for each disease. When users hover over a node, detailed information about each association—including gene, gene location, method, reference tissue, *P*-value, *Z*-value and GWAS summary statistics ID—is shown (Figure 3A). Two customizable features are available to improve user experience. First, users can select different methods to show or hide associations identified by each, allowing for easy confirmation of consistency across methods. Second, users can select specific chromosomes to view detailed information about that region, which can be combined with the first feature for a clearer analysis of disease-related genes.

### Updated online TWAS analysis tool for analyzing GWAS summary statistics

In addition to the precomputed associations and interactive visualization tool available for users, webTWAS 2.0 also offers an updated online TWAS analysis tool (Figure 3B). To lower the barrier to entry for TWAS analysis, the ‘TWAS Online’ page allows users to upload GWAS summary statistics files containing necessary columns such as rsID, effect allele, noneffect allele, *P*-value or *Z*-value. Users can set essential parameters for each method, including tissue reference panels, population, significant thresholds, etc. webTWAS 2.0 now supports four TWAS methods: S-PrediXcan, TWAS-FUSION, sTF-TWAS and 3′aTWAS, with the option to modify the default *P*-value cutoff as needed. To help users track the progress of their analysis jobs, each job is assigned a unique ID. Users can query job progress on the job search page, download results or follow the job’s URL provided via email if they have entered their email address during parameter setup.

## Database construction and improved user interface

webTWAS 2.0 employs Spring Boot for the back-end architecture. On the front end, the interface is built with Vue.js, leveraging Element UI for a smooth user experience. MySQL serves as the database system, allowing for fast querying of GWAS summary statistics and TWAS-associated genes linked to diseases. The TWAS analysis server adopts an asynchronous approach to efficiently manage and schedule user-submitted tasks. All processes are logged and can be tracked through the webTWAS 2.0 dashboard. A detailed system layout is provided in Figure 1.

webTWAS 2.0 features a user-friendly interface and multiple data access options, allowing users to query the database in several steps:

- On the ‘Home’ page, a central search box enables users to easily investigate gene–trait associations. Users can search by diseases of interest, with matching disease entries displayed in a drop-down box. All possible records are shown on the search results page, where each column supports sorting to help users more efficiently find the data of interest. The entire dataset can also be downloaded via a button at the top right of the page. For detailed information about specific associations, users can click on the ‘Reported Trait’ or ‘webTWAS ID’ column to access the detailed page for each disease entry.
- On the ‘Disease’ page, the complete disease entry results of webTWAS 2.0 are displayed. A MeSH tree is available at the top left, allowing users to easily explore diseases of interest. By clicking on a specific disease, users are taken to a detailed gene–trait association page. The top table provides detailed information about the trait, including the ‘Reported Trait’ from the original publication, the mapped ‘MeSH Term’, ‘MeSH ID’, ‘Trait Type’, ‘Trait Description’ and ‘More Information’ hyperlinks to additional details about the disease. In the middle of the page, an interactive Manhattan plot displays all associations for the disease, allowing users to show or hide associations identified by different methods by clicking the corresponding method buttons. For user convenience, the Manhattan plot can be separated by chromosome to facilitate the examination of associated genes in different regions. Hovering over nodes in the Manhattan plot reveals detailed association information, including gene name, gene location, method, tissue reference, *P*-value, *Z*-value and webTWAS ID. The quantile–quantile (Q–Q) plot for visualizing *P*-value distributions for each method is presented below the Manhattan plot. This allows users to assess potential inflation and compare the performance of different methods. Both the Q–Q plot and the Manhattan plot can be downloaded for each method and GWAS summary statistic to facilitate user analysis. Furthermore, all gene associations for each method are available in the database, providing comprehensive access and enabling users to apply customized *P*-value thresholds as needed. The bottom table presents detailed information about each association, which can be freely downloaded by clicking the download button at the top right of the table. Users can also sort columns to browse detailed information about each association more easily (Figure 3C).
- On the ‘Gene’ page, the complete gene entries from webTWAS 2.0 are displayed. The table provides detailed information about each gene, including ‘Gene Symbol’, ‘Ensembl ID’, ‘Location’, ‘Gene Type’, ‘Synonyms’ and ‘Number of Associated Traits’. Blue font entries are clickable, leading to a detailed gene information page, and each column supports sorting to easily find genes of interest. On the detailed gene page, the top table presents additional information about each gene, including a gene description and summary from the NCBI database, gene group and hyperlinks to the gene information pages on the ENSEMBL (47), GeneCards (48) and NCBI (49) databases. The bottom table displays associations related to the gene, with options for downloading the data and more detailed information about these associations (Figure 3D).
- On the ‘Search’ page, users have three options to easily access associations of interest: search by trait, gene or publication. Each search box displays example prompt words to assist users. They can enter information such as trait name, gene name, Ensembl ID, gene location or PMID, and select matching entries from the drop-down box to more efficiently access the associations (Figure 3E).
- On the ‘Downloads’ page, two download options are provided. Users can either enter a disease of interest, select the matching disease entry and click the download button next to the search box, or directly download all associations identified by each method. The table on the page displays detailed information about each download file, including file format, file size and download link (Figure 3F).
- On the ‘TWAS Online’ page, an updated online TWAS analysis tool is provided for users to easily conduct TWAS analyses. The process is divided into two steps: upload the necessary GWAS summary statistics and configure method parameters. For user convenience, example data and demo results are available on the page to help users prepare their data and follow the job process. After uploading the GWAS data, click the ‘Load data’ button. Parameters will be auto-filled in the appropriate fields. Users then need to fill in essential parameters such as sample size, check other necessary parameters and optionally enter an email address to receive notifications about job progress. Afterward, click the ‘Next’ button to proceed to the configuration page. On the configuration page, users can choose the analysis methods, including S-PrediXcan, TWAS-FUSION, sTF-TWAS and 3′aTWAS, and set parameters for each method, such as significance thresholds, statistical models and tissue references. Specifically, MASHR (Multivariate Adaptive Shrinkage in R) (50) is well-suited for integrating data from multiple tissues, allowing for more accurate predictions by borrowing strength from shared SNP effects. This approach is recommended when users have access to multitissue data. Elastic-net is a regularization method that combines LASSO and Ridge regression, making it robust for single-tissue analyses with limited data. This distinction helps users choose based on data complexity and study design. Once all fields are completed, click ‘Submit’ to start the job. A page with a job ID will be generated, allowing users to track the job status. The final analysis results will also be displayed on this page. After the analysis is complete, results will be visualized in a Manhattan plot and also displayed in a table, and the results can be freely downloaded by the user. Alternatively, the ‘Job Search’ page can be used to check the status of a job by entering the job ID.

## Conclusions, limitation and future directions

In the initial version of the webTWAS database, webTWAS 1.0, only 1298 GWAS summary statistics and 3 TWAS methods were included. However, with the development of TWAS methods and the release of large cohorts, the number of GWAS summary statistics and TWAS methods has increased significantly over the past 5 years. The rapid growth of TWAS research has demonstrated its power as a tool for decoding the genetic determinants of complex human diseases, highlighting the need for a more comprehensive TWAS database and an update to webTWAS 1.0. The updated webTWAS 2.0 now includes 7247 GWAS summary statistics of 1588 diseases from 192 publications. It now provides TWAS analysis results from six popular and representative methods, along with interactive visualizations for easy assessment of gene–trait associations. Additionally, a user-friendly online TWAS analysis tool has been introduced to facilitate these analyses. These resources are valuable for further exploration of genetic determinants of human complex diseases, discovery of disease targets and promotion of research. While multiple resources are available for GWAS, such as GWAS Catalog (37), GWASDB (51), GWAS Atlas (52), CAUSALdb (34) and GRASP (39), TWAS resources have been limited to TWAS-hub, TWAS Atlas and webTWAS. webTWAS 2.0 addresses this gap in comprehensive TWAS resources and aims to facilitate TWAS analysis. For performance, the average number of significant genes per tissue for S-PrediXcan in webTWAS 2.0 is 11.9, similar to TWAS Atlas at 10.8. For TWAS-FUSION, webTWAS 2.0 shows an average of 10.8 significant genes per tissue, higher than TWAS-hub at 5.5 but less than TWAS Atlas at 15.6. These results demonstrate that webTWAS 2.0 performs comparably to other TWAS platforms while providing a significantly larger dataset and incorporating a wider range of TWAS methods.

Although webTWAS 2.0 integrates six TWAS methods, several others are not yet included in the platform, particularly those that use kernel machines (5,9,10,53) for the second step of association analysis, which requires access to genotype data. In webTWAS 2.0, we have integrated the multitissue TWAS method UTMOST to leverage the advantages of using multiple tissues in the analysis, improving both power and accuracy. Associations for all tissues have been calculated using S-PrediXcan, providing comprehensive data access and allowing users to select their preferred tissues. In future versions of webTWAS, we plan to integrate additional multitissue methods, such as TisCoMM (8), to further improve the platform’s capability for robust gene–trait association studies using multiple tissue types. One shortcoming of traditional TWAS methods is their inability to account for uncertainty in the imputed gene expression, which can lead to reduced statistical power and less reliable results. To address this, future updates of webTWAS will integrate the CoMM series TWAS methods (8,54–56), which provide a more sophisticated framework by incorporating this uncertainty directly into the analysis. The CoMM methods, particularly CoMM-S2 (55), offer better precision and statistical power by jointly modeling both gene expression imputation and association with traits. Moving forward, it will be important to consider incorporating genotype data alongside GWAS summary statistics for more comprehensive TWAS analyses. Currently, webTWAS 2.0 only includes GWAS data for the EUR population due to limitations in available reference panels. To address this, we plan to regularly update the database with new GWAS data and TWAS methods. As larger cohorts become available, future updates will incorporate GWAS data from non-EUR populations and additional TWAS methods to ensure broader applicability and greater accuracy.

## Data Availability

The data underlying this article are available in webTWAS 2.0 (http://www.webtwas.net) and can be freely downloaded.
